# Quantitative morphometric analysis reveals morphological overlap among traditional sepal types in *Cymbidium goeringii*

**DOI:** 10.3389/fpls.2026.1947160

**Published:** 2026-10-28

**Authors:** Yongqi Zhi, Yi Yang, Zishan Ahmad, Hu Zhao, Bo Fu, Fengjun Wu, Lili Wang, Qiang Wei, Sen Wang

**Affiliations:** 1 Institute of Biotechnology, Shaoxing Academy of Agricultural Sciences, Shaoxing, China; 2 State Key Laboratory of Tree Genetics and Breeding, Co-Innovation Center for Sustainable Forestry in Southern China, Bamboo Research Institute, Nanjing Forestry University, Nanjing, China; 3 Yinlanquan Flower and Tree Farm, Shaoxing, China

**Keywords:** *Cymbidium goeringii*, floral architecture, floral morphometrics, ornamental orchid, sepal-shape classification

## Abstract

**Introduction:**

Traditional sepal classification in *Cymbidium goeringii* relies on expert-based descriptions, but the quantitative basis and extent of morphological overlap among categories remain poorly understood. This study investigated the planar morphometric differentiation of traditional sepal categories.

**Methods:**

A total of 1,380 floral-organ observations from 46 traditional cultivars were collected using a standardized scanning protocol. Morphometric descriptors were analyzed using linear discriminant analysis (LDA), with SMOTE balancing and stratified cross-validation to explore morphological separation among categories.

**Results:**

Shui-xian-ban showed substantial morphological overlap with mei-ban, whereas zhu-ye-ban and he-ban exhibited relatively greater morphological separation. Sepal tip angle and sepal length contributed most strongly to the observed separation. Organ-specific misclassification patterns also highlighted the limitations of two-dimensional representation for capturing three-dimensional floral features such as qi-dou.

**Discussion:**

The findings indicate that traditional sepal categories do not consistently correspond to distinct planar morphometric boundaries. Quantitative morphometrics provides an exploratory approach to characterizing morphological variation associated with traditional floral classifications, but the results do not establish a validated cultivar-authentication method. Independent cultivar-level and external validation across additional germplasm collections are needed to assess generalizability and practical applicability.

## 1 Introduction

*Cymbidium goeringii* (Rchb.f.) Rchb.f., commonly known as the spring orchid, is not merely an ornamental species; it is a living cultural heritage that has shaped Chinese aesthetic consciousness for over a millennium (Hew and Wong, 2023). Central to its appreciation is the ban-xing (petal-shape) classification system, which divides cultivars into four canonical categories: he-ban (lotus-petal shape, broad and rounded), mei-ban (plum-petal shape, rounded head with moderate length), shui-xian-ban (narcissus-petal shape, elongated with a hooded center), and zhu-ye-ban (bamboo-leaf shape, slender and acuminate) (Jin et al., 2023). This traditional system, which has its roots in past orchid cultivation techniques, is still a crucial part of horticulture knowledge and cultivar appraisal. The degree to which these groups’ traditional morphological boundaries correlate to numerically discernible patterns of floral shape are still unclear, though, as these classifications are mostly established by expert visual judgement. The digitization of germplasm resources has become a global imperative for non-model horticultural crops (Engels and Ebert, 2021; Smyth et al., 2020). For *C. goeringii*, molecular markers such as SSR and SNP have been widely applied for cultivar identification and genetic diversity analysis, supporting precise genetic fingerprinting in breeding programs (Nam et al., 2023). However, the phenotypic traits that are used to identify and value traditional orchid cultivars are not readily quantified by genetic identity. Therefore, morphological descriptors offer an additional level of information, especially for conventional classifications that rely more on visible floral architecture than genetic identity.

Two-dimensional (2D) morphometrics provides an effective intermediate framework for linking quantitative shape analysis with traditional morphological terminology, offering low-cost and interpretable phenotypic descriptors (Montanaro et al., 2023). While still being somewhat interpretable in comparison to end-to-end deep-learning techniques, such descriptors can offer more reproducibility than simply subjective visual evaluation. While applications to conventional ornamental germplasm are still relatively limited, quantitative image-based phenotyping has been applied more frequently to agricultural characteristics and plant organs (Jiang and Li, 2020; Murphy et al., 2024). Crucially, the goal of using morphometrics in this situation is to measure the morphological variation that underlies existing descriptive categories and identify areas where those categories exhibit continuous overlap or distinct separation rather than to replace conventional expert knowledge. Additionally, ornamental flowers may have intricate three-dimensional structures that planar photographs cannot fully capture, which poses a significant challenge to 2D methods (Yang et al., 2026).

Prior research has demonstrated that morphometric techniques and quantitative image analysis can be used to characterise horticultural materials, distinguish phenotypic groups, and define plant-organ form. Therefore, neither the broad idea of quantitative morphometrics nor even the application of traditional statistical classification in general constitutes the novelty of the current study. Instead, the biological novelty is found in the application of quantitative shape analysis to a floral classification system in *C. goeringii* that is culturally established but quantitatively unresolved, with an emphasis on determining whether traditionally recognised sepal types represent distinct morphological groups or partially overlapping regions in the space of floral shape. As a result, the methodological novelty is pragmatic and limited: we combine discriminant analysis, contour-derived descriptors, standardised sepal imaging, and feature evaluation to produce an understandable framework that connects quantifiable shape qualities to conventional horticultural nomenclature. The application novelty lies in translating this quantitative framework to traditional *C. goeringii* germplasm, where morphological appraisal is a significant constituent of cultivar recognition and where three-dimensional floral features might not be captured completely by conventional planar descriptors. This distinction is crucial because neither a novel morphometric algorithm nor a genuinely unique statistical method are intended to be proposed in this study. Instead, it contributes by offering a quantitative evaluation of a well-established expert classification system and identifying the quantifiable features of sepal form that align with or deviate from conventional morphological ideas. In particular, determining whether shui-xian-ban represents a discrete category or a transitional region between other sepal types carries biological and horticultural significance as it directly addresses the morphological boundaries implied by traditional classification. This distinction is crucial because neither a unique morphometric algorithm nor a genuinely novel statistical approach are the goals of the current investigation. Instead, it contributes by offering a quantitative evaluation of a well-established expert classification system and identifying the quantifiable characteristics of sepal form that align with or deviate from conventional morphological ideas. In particular, determining whether shui-xian-ban represents a discrete category or a transitional region between other sepal types carries biological and horticultural significance as it directly addresses the morphological boundaries implied by traditional classification.

Therefore, the quantitative characterisation of sepal-shape variation in *C*. *goeringii* is the main emphasis of this study. We specifically assess the degree of morphological separation and overlap between the four conventional categories rather than assuming that they are intrinsically distinct. In all, 1380 sepals from 46 traditional cultivars were analyzed by using standard image acquisition procedures and a set of contour-based morphometric descriptors. The analysis was performed in order to address the following questions: (1) which of the quantitative descriptors contribute most to the differentiation between the traditional sepal categories; (2) how much can be the traditional categories differentiated with interpretable morphological variables; (3) which of the categories are substantially overlapping indicating possible transitional forms; and (4) which misclassifications suggest limitations of planar morphometric representations for capturing three-dimensional features such as qi-dou.

Rather than claiming a globally recognized cultivar-classification or authentication method, the study seeks to establish a quantitative description of conventional floral variety. The resulting framework bridges the gap between measurable floral morphology and expert-based horticultural terminology. It also highlights the limitations that need to be addressed by future research that incorporates independent cultivar-level validation, three-dimensional imaging, and more extensive germplasm sampling.

## 2 Materials and methods

### 2.1 Plant materials and image acquisition

46 traditional cultivars of *C. goeringii*, representing the four canonical sepal-shape categories, were selected from the germplasm collection at Shaoxing Agricultural Science Research Institute and Yinlanquan Flower and Tree Farm in Keqiao District, Shaoxing City (**Supplementary Table 1**). The sampled material represented traditional horticultural cultivars maintained predominantly through vegetative division and therefore generally corresponding to clonal lineages. The number of flowers sampled for each cultivar was not equal since the availability of particular cultivars varied significantly. More flowers were given by cultivars that were easier to replicate or more prevalent in cultivation, while less flowers were contributed by cultivars that were harder to propagate or represented in culture. To maintain the morphological heterogeneity represented in the accessible germplasm and prevent disproportionate representation of uncommon cultivars, this unequal availability was maintained rather than enforcing equal sampling among cultivars. The number of flowers sampled from each cultivar was recorded in the sampling dataset. The four sepal-shape categories were based on the pre-existing traditional horticultural classification assigned to each cultivar within the source germplasm collections. Thus, the category labels used in this study represent established cultivar-level classifications rather than classifications newly assigned to individual flowers or sepals during the present experiment. All plants were grown under uniform cultivation conditions in a semi-shaded greenhouse (photosynthetic photon flux density: 80-120 µmole m^-2^s^-1^; temperature: 18-25 °C; relative humidity: 60-80%) to minimize environmental plasticity. During the peak flowering period (February-March), fully opened and undamaged flowers were selected. For outline acquisition, the two lateral petals and the three outer tepals-two lateral sepals and one center sepal-were carefully separated and placed separately on a flatbed scanner. Each flower therefore contributed five floral organs, while the specialized central lip (labellum) was excluded. Every organ was arranged as uniformly as possible along its longitudinal axis, without purposeful folding or stretching. A metric scale was included in the scanning field for spatial calibration. The same scanning setup and positioning procedure were used for all samples. A total of 1,380 floral-organ observations passed quality control.

Instead of estimating population-level variation or performing cultivar authentication, the current study was intended to characterize morphological variation connected with recognized traditional cultivar categories. The number of biological replicates per cultivar was not standardized since the plants that were available constituted horticulturally maintained material with uneven representation among cultivars. As a result, rather than being independent biological replicates reflecting distinct genotypes, individual floral parts were handled as morphological observations for descriptive and exploratory analyses.

All sampled flowers and sepals from a certain cultivar inherited the corresponding established cultivar-level category since the traditional classification is upheld at the cultivar level. Since the current study did not independently reclassify individual flowers or sepals, the four category labels should be considered conventional horticulture reference labels rather than independently verified experimental ground truth. This distinction is crucial because, rather than developing a new expert-based grading system, the study’s goal was to quantify morphological variation associated with known traditional categories, including their degree of overlap. Since the answer categories were obtained from the established cultivar identities and traditional classifications of the source collections rather than being allocated *de novo* to particular floral organs, neither blinded expert evaluation nor inter-rater scoring was carried out during image gathering.

### 2.2 Contour feature extraction

External contours of the five sampled floral organs (three sepals and two lateral petals) were extracted using a custom image-processing pipeline developed in MATLAB (R2016b, MathWorks, Natick, MA, USA) (Klein and Svoboda, 2017), following the geometric morphometric framework for outline-based shape analysis. Recent studies of plant-derived biological materials have similarly emphasized systematic analytical workflows and detailed reporting of processing and structural-characterization procedures to improve methodological transparency and reproducibility (Gong et al., 2024). The workflow comprised: (1) RGB-to-grayscale conversion; (2) global Otsu thresholding; (3) morphological closing; (4) boundary tracing via the Moore-neighbor algorithm; and (5) coordinate extraction at equal angular intervals. Only the target floral-organ contour was kept for further analysis; the scale reference and image background were not included in the contour selection procedure. All images were subjected to the identical preprocessing and contour-extraction settings. For each contour, the following primary variables were computed: scan. length (cm), scan. width (cm), LWR_ obs (dimensionless), width_ pos_ ratio_ obs (dimensionless), tip_ angle (degrees), R. square (dimensionless, 0-1), and sample. size (integer). The coefficient of determination achieved by fitting the extracted contour to the relevant reference geometric model was represented by contour R2, and the width-position ratio was computed as the relative longitudinal position of maximal contour width. Taper ratio and relative tip width were calculated from the relative width of the distal region and were used only in the exploratory Model 4 analysis. All spatial measurements were calibrated against the metric scale included in the scanning field. Elliptic Fourier descriptors (EFDs) were additionally calculated as a supplementary exploratory analysis to characterize contour-frequency information not represented by the primary geometric descriptors. The final LDA model and model selection did not use EFD results; instead, they were used descriptively to investigate variations in contour complexity. Only a portion of the overall floral phenotype is captured by the extracted descriptors, which represent planar geometric characteristics of sepal outline; characteristics involving three-dimensional curvature, texture, pigmentation, surface features, or physiological and biochemical properties were outside the purview of the current analysis. The obtained descriptors reflect two-dimensional geometric features rather than the entire three-dimensional flower architecture since the image-based analysis only recorded the external planar contour of detached floral parts. In particular, curvature, hooding, folding, and other spatial characteristics that depend on organ thickness or orientation were not directly quantified. No claim of full three-dimensional structural characterization is made because the organs were separated and placed on a planar scanner, which results in measurements that represent two-dimensional projected geometry and may not fully maintain curvature-dependent features.

### 2.3 Statistical design

The response variable in the classification study was the established traditional floral category of the source cultivar (he-ban, mei-ban, shui-xian-ban, or zhu-ye-ban). All floral organs sampled from a particular cultivar inherited the associated conventional category label since these traditional categories are assigned at the cultivar level. Therefore, the categorization does not represent an independent reassignment or authentication of particular cultivars; rather, it assesses the degree to which the measured two-dimensional morphology of the sampled floral organs corresponds to the pre-existing traditional classifications. Initially, a stratified 80:20 split was used to separate the dataset into training and held-out test subsets. The held-out test subset was kept completely untouched during model development. Recent computational identification studies have demonstrated the value of systematic feature extraction and quantitative classification for improving objective identification accuracy (Wang et al., 2026). In the present study, a similarly quantitative strategy was adopted by combining predefined morphometric descriptors with variance-inflation-based feature screening, stratified cross-validation, and independent test-set evaluation to assess the discriminatory performance of the LDA framework. Only the matching training section of each cross-validation iteration was used for all data-dependent preprocessing and SMOTE within the training data; the validation fold was left unaltered until prediction (Chawla et al., 2002). SMOTE generates synthetic minority-class samples by linearly interpolating between each minority instance and its K nearest neighbors in feature space (K = 3). Linear discriminant analysis (LDA) (Fisher, 1936) was performed using the MASS package in R (v4.3, R Core Team, 2023). Prior probabilities were fixed at 0.25 for each class to minimize prevalence-driven bias. Because the predictors were continuous morphometric descriptors and the resulting discriminant functions allow for direct interpretation of the contribution of individual morphological traits, LDA was chosen as an exploratory, interpretable framework for quantifying separation among the four conventional categories. For complex visual and structural characterization, recent classification studies have shown the benefits of combining complementary information sources and sophisticated computational techniques. These include multimodal AI frameworks that integrate visual data with contextual knowledge and image-analysis workflows that quantitatively characterize complex structures (O’Brien et al., 2026; Xing et al., 2025). Instead of using a multimodal or deep-learning classifier in the current investigation, we purposefully kept an interpretable morphometric framework based on clearly stated geometric descriptors. Establishing that the four categories represent statistically or physiologically distinct populations was not the goal of the investigation. LDA has proven effective for morphological classification in plant species with continuous trait variation, including forest trees (James et al., 2013), supporting its application to the *C. goeringii* sepal-shape continuum. The present analysis used a fixed SMOTE K value, and sensitivity to alternative K values was not systematically evaluated; therefore, the robustness of synthetic-sample generation to the choice of K remains to be established in future analyses.

Model performance was evaluated through two complementary validation strategies. First, for independent test-set validation, the dataset was randomly partitioned into 80% training and 20% testing subsets using stratified sampling implemented by the create Data Partition function in the caret package (Cohen, 1960). Second, five-fold stratified cross-validation (CV) was performed by dividing the dataset into five approximately equal folds while preserving class proportions. Classification performance was assessed using overall accuracy and Cohen’s Kappa coefficient (Cohen, 1960), which were averaged across the five validation folds. The 1,380 observations are morphological rather than completely separate biological replicates because several floral organs were taken from the same flowers and cultivars. Therefore, rather than being formal population-level inference, organ-level statistical studies were regarded as exploratory evaluations of morphological variation linked to traditional categories. Future research employing balanced sampling designs and mixed-effects techniques should take into account the hierarchical effects at the cultivar, plant, and flower levels that were not specifically addressed in this study.

### 2.4 AB testing for variable robustness

To ensure that our discriminant functions rested on biologically meaningful rather than technically confounded signals, we designed a four-model AB comparison (**Table 1**). Model 1 (M1, baseline): included all six original features. Model 2 (M2, no sample size): excluded sample. size. Model 3 (M3, point density): replaced sample size with point density = sample. size/scan. length. Point density was considered a processing-dependent geometric descriptor rather than a direct indicator of biological contour complexity since boundary-point count can be affected by image resolution, contour-tracing parameters, and resampling techniques. Therefore, better classification performance was not seen as proof that point density is an independent biological attribute; rather, the comparison of M1–M4 was largely interpreted as a sensitivity analysis of descriptor choice. Model 4 (M4, taper proxies): added taper_ ratio and relative_ tip_ width. Multicollinearity among predictors was assessed using variance inflation factors (VIF), with a threshold of 5.0; VIF was used specifically to diagnose predictor redundancy and was not considered a test of the broader assumptions of LDA (James et al., 2013). Variance inflation factors (VIFs) were computed for the potential morphometric descriptors in order to reduce multicollinearity and enhance the stability and interpretability of the LDA discriminant functions. Scan length and scan width were used to calculate the derived descriptor LWR_obs, which exhibited significant multicollinearity with these initial values (VIF > 400). Therefore, LWR_obs was excluded from subsequent LDA modeling. Additionally removed from the predictor set were the prediction-derived variable (pred) and scan area, which was deemed redundant with the linear size measurements. All of the predictors that were kept after iterative variable screening had VIF values less than 5. VIF was only used to evaluate predictor redundancy and was not regarded as a test of the more general principles of LDA. Model selection was based primarily on 5-fold CV Kappa together with multicollinearity diagnostics; held-out test-set performance was not used for model selection. However, if the highest-Kappa model exhibited severe multicollinearity (VIF > 5), we advocated the best-performing alternative that satisfied the independence assumption.

**Table 1:** Descriptive statistics of seven morphometric variables across the four traditional sepal-shape categories of *Cymbidium goeringii*.

Trait	He-ban	Mei-ban	Shui-xian-ban	Zhu-ye-ban	F-value	P-value
Scan length (cm)	2.352 ± 0.481a	2.510 ± 0.494a	2.703 ± 0.609b	3.445 ± 0.616c	142.3	<0.001
Scan width (cm)	1.261 ± 0.261a	1.203 ± 0.163a	1.128 ± 0.187b	1.015 ± 0.139c	58.7	<0.001
Length-to-width ratio (LWR_obs)	1.877 ± 0.237a	2.095 ± 0.354b	2.430 ± 0.545c	3.473 ± 0.879d	231.5	<0.001
Tip angle (°)	81.94 ± 10.65a	70.54 ± 11.03b	62.86 ± 12.07c	50.08 ± 10.86d	178.2	<0.001
Width-position ratio	0.437 ± 0.068b	0.392 ± 0.062a	0.400 ± 0.069a	0.464 ± 0.089b	24.6	<0.001
Contour R^2^	0.985 ± 0.010b	0.992 ± 0.004a	0.991 ± 0.004a	0.985 ± 0.011b	31.4	<0.001
Boundary-point count (n)	777.3 ± 147.8a	797.1 ± 134.9a	818.5 ± 155.4a	966.2 ± 142.1b	45.2	<0.001

Values are mean ± SD. Different lowercase letters indicate significant differences among categories according to Tukey’s HSD test (P < 0.05). Because multiple floral organs were sampled from the same flowers and cultivars, these organ-level comparisons are presented as exploratory descriptions of morphometric variation rather than as tests based on independent biological replication.

### 2.5 Software and reproducibility

All statistical analyses and visualizations were conducted in R v4.3 (R Core Team, 2023). Key packages included: MASS for LDA; caret for data partitioning and cross-validation; smote family for SMOTE resampling; dplyr and tidyr for data manipulation; and ggplot2 for graphics (Wickham et al., 2023). The raw morphometric dataset, supplementary tables and figures, metadata, and analytical information supporting the findings of this study are available from the corresponding author upon genuine request. On request, the statistical analysis scripts and MATLAB image processing methodology may also be provided.

## 3 Results

### 3.1 Descriptive morphometrics across four sepal-shape categories

Descriptive differences among the traditional sepal-shape categories were observed for all primary morphometric traits (**Table 1**; **Figure 1**). Organ-level ANOVA detected significant differences among categories (P < 0.001); however, because multiple organs originated from the same flowers and cultivars, these P values should be interpreted cautiously and regarded as exploratory evidence of morphometric differentiation rather than as formal population-level inference. Zhu-ye-ban exhibited the greatest mean sepal length (3.445 ± 0.616 cm) and the highest length-to-width ratio (LWR = 3.473 ± 0.879). He-ban displayed the greatest width (1.261 ± 0.261 cm) and the lowest LWR (1.877 ± 0.237). Tip angle decreased monotonically from he-ban (81.94° ± 10.65°) to mei-ban (70.54° ± 11.03°), shui-xian-ban (62.86° ± 12.07°), and zhu-ye-ban (50.08° ± 10.86°) (**Supplementary Figure S1**). This gradient quantitatively validates the traditional distinction between ‘tou-yuan’ (rounded head, high angle) and ‘jian-chang’ (slender tip, low angle).

**Figure 1:**
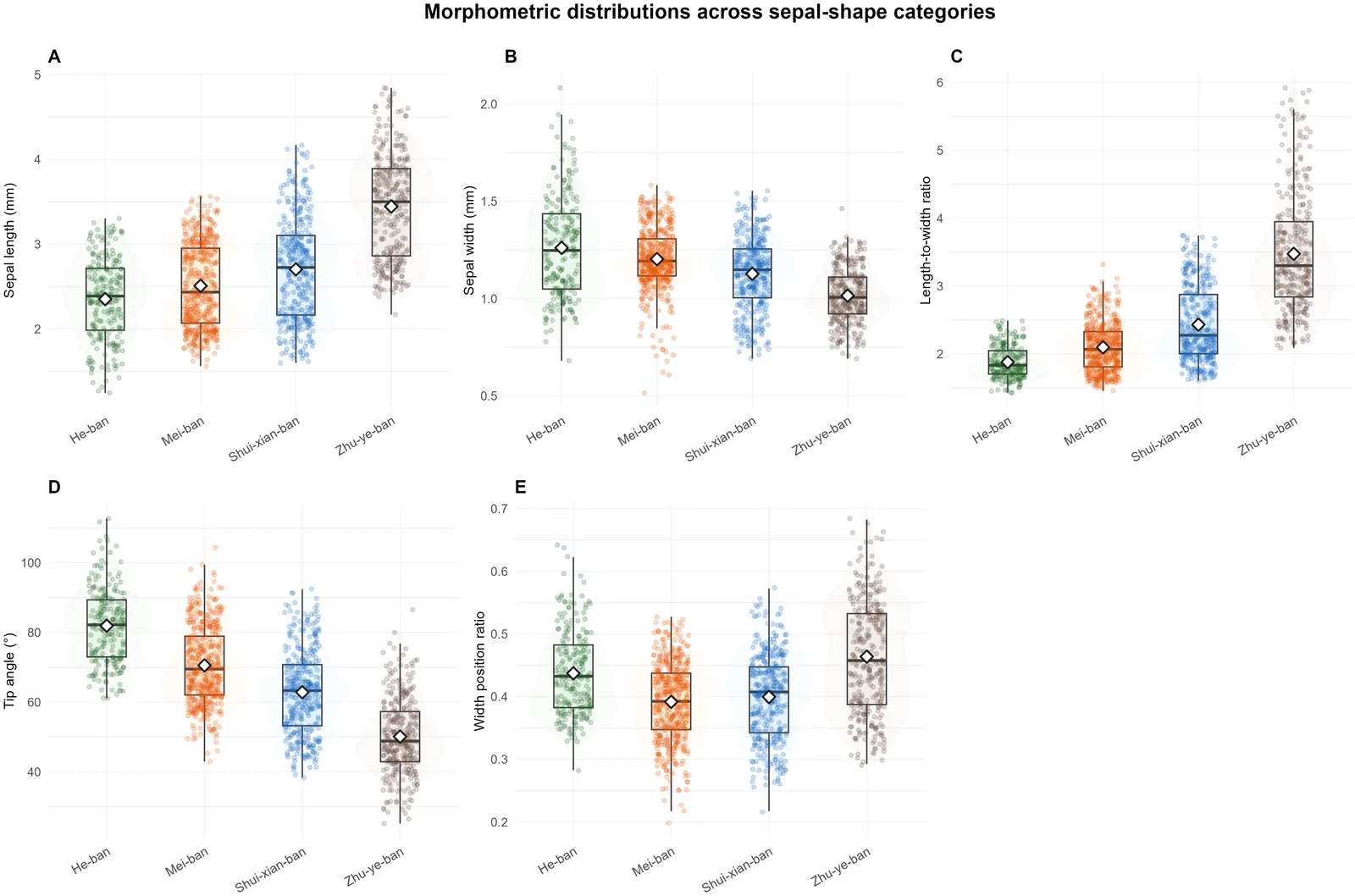
Morphometric differentiation of traditional sepal - shape categories in *Cymbidium goeringii*. Violin-box plots show significant differences among he-ban, mei-ban, shui-xian-ban, and zhu-ye-ban for **(A)** Sepal length (cm). **(B)** Sepal width (cm). **(C)** Length-to-width ratio (LWR). **(D)** Tip angle. **(E)** Width-position ratio. Each panel shows violin density plots (light shading), boxplots (dark boxes with median line), individual data points (jittered dots), and group means (white diamonds). Color coding: he-ban (green), mei-ban (orange), shui-xian-ban (blue), zhu-ye-ban (brown). Different lowercase letters denote significant differences among categories (Tukey’s HSD, P < 0.05).

### 3.2 Multicollinearity diagnosis

VIF analysis of the baseline model (M1) revealed catastrophic multicollinearity between two size-related variables: scan. length (VIF = 70.63) and sample. size (VIF = 69.97) (**Supplementary Figure S2**; **Supplementary Table S2**). Both values exceeded the conventional threshold of 5.0 by more than an order of magnitude. This near-perfect collinearity is mechanistically expected: at fixed scanning resolution, larger sepals yield both longer Feret diameters and more boundary pixels. The remaining predictors (scan. width, width_ pos_ ratio_ obs, tip_ angle, R. square) showed acceptable VIF values (1.42-6.21), indicating no concerning redundancy among the non-size variables.

### 3.3 Classification performance: the AB test

The four-model comparison revealed a trade-off between predictive performance and statistical robustness (**Supplementary Table S3**; **Supplementary Figure S3**). The baseline model (M1) achieved the highest 5-fold CV Kappa (0.4502 ± 0.0760) and the highest test-set balanced accuracy (0.7098). However, its severe multicollinearity rendered its coefficient estimates unstable. Model 2, which excluded sample. size, exhibited a modest but clear performance degradation (CV Kappa = 0.4190 ± 0.0851), confirming that sample. size contributed genuine discriminative signal beyond mere size scaling.

Model 3, which substituted sample size with point density, showed comparable classification performance, indicating that the normalized descriptor retained information associated with the traditional categories. However, this result was viewed as descriptor-level robustness rather than proof of biological contour complexity because point density is still potentially sensitive to picture resolution and contour-processing parameters. Its balanced accuracy (0.7106) was comparable to M1. Given the substantial multicollinearity of M1 and the small difference in cross-validation Kappa between M1 and M3, M3 was retained as the more statistically robust model for exploratory interpretation. Model 4, incorporating taper_ ratio and relative_ tip_ width, did not improve overall performance (CV Kappa = 0.4212) and exhibited severe multicollinearity (taper_ ratio VIF = 22.02, relative_ tip_ width VIF = 35.12), indicating that the two-dimensional taper proxies that were examined did not enhance discrimination and might not accurately reflect morphological traits that planar outline measures are unable to capture.

All models substantially exceeded the no-information rate (0.3504), demonstrating that the morphometric signal was genuine rather than random. M3 was kept as the recommended descriptor configuration for exploratory interpretation since M1 and M3 demonstrated extremely similar prediction performance while M1 displayed severe multicollinearity. Since M1 was the model utilised for the first class-level error analysis, the class-specific confusion matrix that follows is predicated on it; as a result, these results are understood as descriptive rather than as validation of M3.

### 3.4 Class-specific performance and error patterns

The confusion matrix of the baseline model (M1) on the independent test set (n = 274) revealed asymmetric misclassification patterns (**Table 2**; **Figure 2**). Zhu-ye-ban was the most accurately recognized category (72.58% correct), followed by mei-ban (60.67%) and he-ban (54.24%). Shui-xian-ban exhibited the poorest recognition (39.06% correct), with 35.94% of shui-xian-ban instances misclassified as mei-ban and 18.75% as zhu-ye-ban. Conversely, only 6.15% of he-ban and 3.28% of zhu-ye-ban were misclassified as shui-xian-ban, indicating substantial overlap of *shui-xian-ban* with neighboring categories in the measured two-dimensional morphometric space. Representative examples of correctly and incorrectly classified specimens illustrate the major sources of classification error and the associated morphometric characteristics (**Supplementary Table S4**).

**Table 2:** Classification performance of the baseline LDA model (M1) on the independent test set and 5-fold cross-validation.

A. Overall performance metrics					
Metric		Test set		5-fold CV (mean ± SD)	
Accuracy		0.5693		0.5934 ± 0.0544	
Kappa		0.4173		0.4502 ± 0.0760	
Balanced accuracy		0.7098		–	
No-information rate		0.3504		–	
B. Class-specific metrics (test set)					
Class	Sensitivity	Specificity	Precision	F1	Prevalence
He-ban	0.5424	0.9442	0.7273	0.6214	0.2153
Mei-ban	0.6067	0.7730	0.5625	0.5838	0.3248
Shui-xian-ban	0.3906	0.7762	0.3472	0.3676	0.2336
Zhu-ye-ban	0.7258	0.9198	0.7258	0.7258	0.2263
Macro-average	0.566	0.8533	0.591	0.575	–

Sensitivity = true positive rate; Specificity = true negative rate; Precision = positive predictive value; F1 = harmonic mean of sensitivity and precision. Macro-average = unweighted mean across all classes.

**Figure 2:**
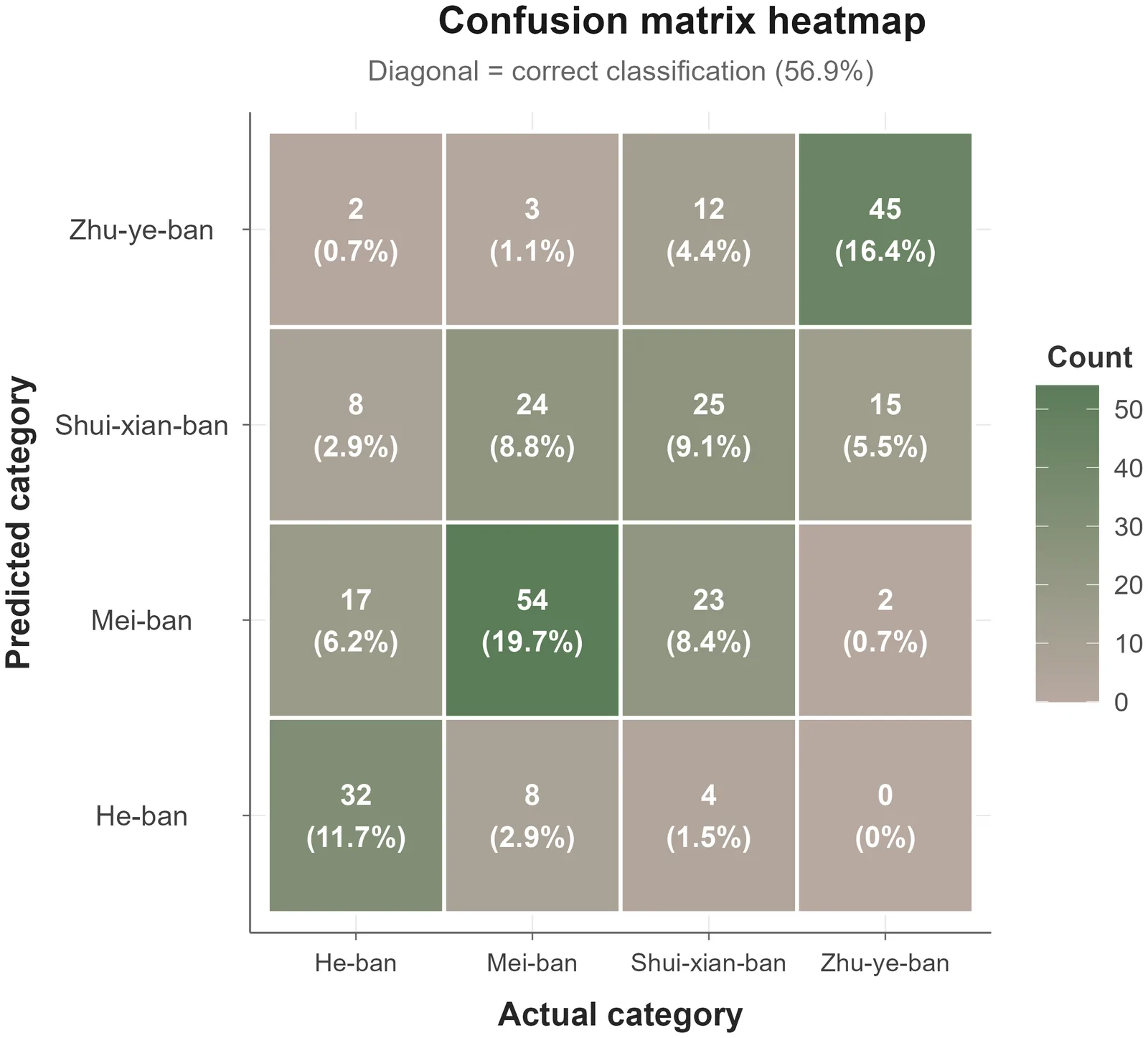
Confusion matrix reveals asymmetric overlap between shui-xian-ban and mei-ban. Heatmap of the baseline LDA model (M1) on the independent test set (n = 274). Zhu-ye-ban shows the highest recognition accuracy, whereas shui-xian-ban is frequently misclassified as mei-ban (35.9%), indicating a continuous morphological transition rather than a discrete category boundary.

### 3.5 Organ-specific classification reliability

*Post-hoc* analysis of misclassified samples by floral organ type revealed striking organ-dependent error patterns. In the test set, the central sepal (zhu-ban, suffix -1) exhibited the lowest error rate (35.5%), whereas the lateral petals (peng-ban, suffix -4 and -5) showed the highest error rates (51.7% and 43.9%, respectively). The lateral sepals (fu-ban, suffix -2 and -3) occupied intermediate positions (39.5% and 44.4%). This gradient—central sepal < lateral sepal < petal - is consistent with the classical maxim ‘zhong-gong ding ban-xing’ (the central sepal determines the petal shape).

More importantly, the misclassification direction of shui-xian-ban was organ-specific (**Supplementary Figure S4**). The lateral sepals (fu-ban, -2 and -3) predominantly drifted toward zhu-ye-ban (68.8% of shui-xian-ban errors from lateral sepals), consistent with their elongated, slender 2D outlines (mean LWR: 2.80 ± 0.50). Conversely, the petals (peng-ban, -4 and -5) predominantly misclassified as mei-ban (69.6% of shui-xian-ban petal errors), because their 2D projections exhibit rounded, obtuse-tip outlines (mean tip angle: 73.2° ± 8.7°) geometrically similar to mei-ban petals (mean tip angle: 79.6° ± 10.2°).

Morphometric ANOVA confirmed significant organ-type differences within shui-xian-ban (tip angle: F_4,359_ = 88.8, P < 0.001; LWR: F_4,359_ = 85.9, P < 0.001), with descriptive statistics summarized in **Supplementary Table S4**. The magnitude of within-cultivar organ divergence rivals that of between-cultivar differences, suggesting that any practical classification system must account for organ type rather than treating all floral organs as exchangeable replicates.

### 3.6 Feature importance and biological interpretation

The first two linear discriminants (LD1 and LD2) collectively explained 89.4% of the among-group variance (LD1: 62.7%; LD2: 26.7%). Standardized structure coefficients (**Table 3**; **Figures 3**, **4**) showed that tip_ angle dominated LD1 (structure coefficient = 0.821, accounting for 40.1% of LD1’s discriminative power), followed by scan. length (-0.720, 30.8%). This indicates that LD1 primarily separates acute-tipped, elongated sepals (zhu-ye-ban, shui-xian-ban) from obtuse-tipped, compact sepals (he-ban, mei-ban). On LD2, width_ pos_ ratio_ obs (0.524, 24.2%) and R. square (-0.664, 38.8%) were the leading contributors, distinguishing flowers with distal width maxima and irregular contours from those with basal maxima and smooth elliptical outlines. Consistent with these findings, the supplementary EFD analysis indicated relatively greater high-frequency contour variation in zhu-ye-ban, providing additional descriptive evidence of differences in planar outline complexity. This discovery was interpreted descriptively rather than as an independently validated classification feature since EFDs were not included in the final classifier. The supplementary EFD analysis did not provide sufficient evidence to justify replacing the simpler contour-based descriptors used in the primary analysis (**Supplementary Figure S5**).

**Table 3:** Structure coefficients of the six morphometric predictors for LD1.

Variable	Structure coefficient	Contribution (%)
Tip angle	0.821	40.08
Scan Length	-0.720	30.79
Boundary-point count	-0.506	15.21
Scan Width	0.436	11.30
Width-position ratio	-0.206	2.52
Contour R^2^	0.056	0.20

Structure coefficients represent the correlations between each predictor and LD1 and are ordered by absolute magnitude. The squared structure coefficient was divided by the total of the squared structure coefficients for each predictor to determine the normalised squared structure coefficient, which was then represented as a percentage. It should not be taken as an inferential effect size, percentage of variance explained, or direct biological contribution to classification; rather, it is offered as a descriptive assessment of each predictor’s relative correlation with LD1. Due to its extreme multicollinearity with these initial measurements (VIF > 400), LWR_obs—a derived descriptor derived from scan length and scan width—was not included in the LDA. Consequently, the stated discriminant model did not include LWR_obs among its predictors.

**Figure 3:**
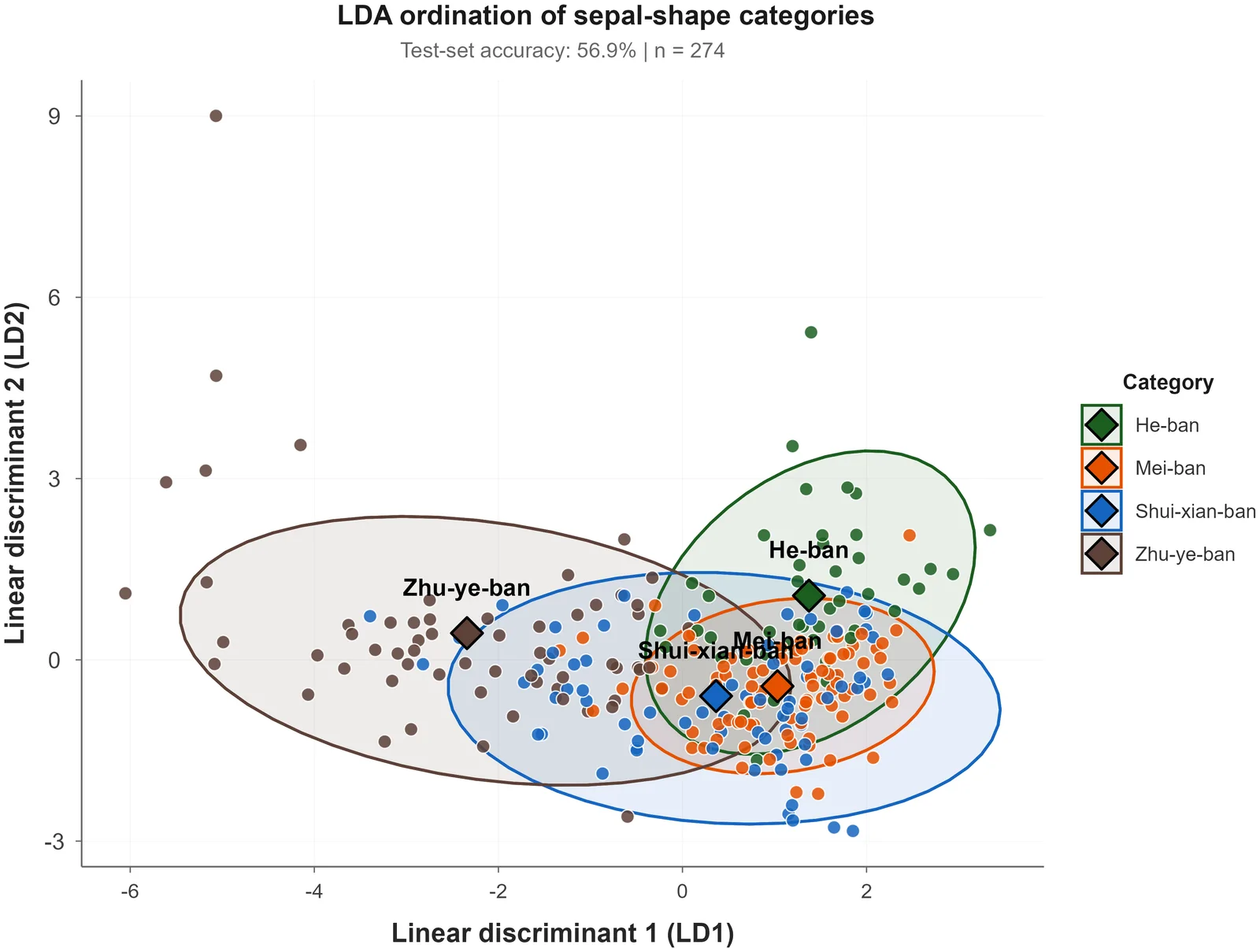
LDA ordination of sepal-shape categories in morphometric space. LDA ordination of sepal-shape categories in the linear discriminant space (test set, n = 274). Data points are colored by actual category; 95% confidence ellipses delineate group boundaries. Black diamonds mark group centroids with English category labels. LD1 (62.7% of among-group variance) primarily separates acute-tipped elongated sepals (left) from obtuse-tipped compact sepals (right). LD2 (26.7% of variance) distinguishes distal-width-maximum sepals (top) from basal-width-maximum sepals (bottom). Note the overlap between shui-xian-ban and mei-ban ellipses.

**Figure 4:**
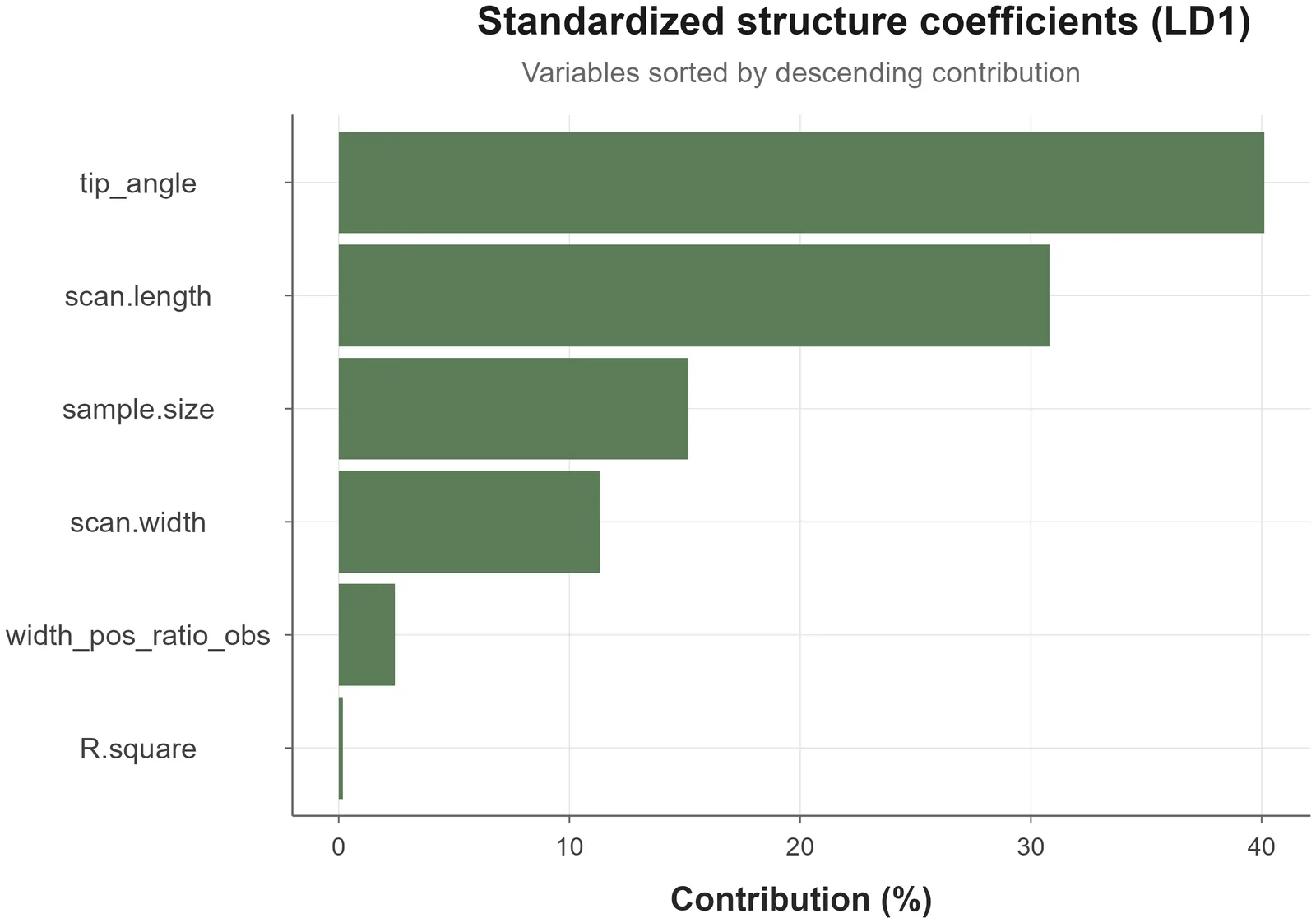
Tip angle is the dominant contributor to sepal-shape discrimination. Standardized structure coefficients of the six morphometric variables on Linear Discriminant 1 (LD1). Variables are sorted by descending absolute contribution. Tip angle dominates LD1 (40.1% contribution, structure coefficient = 0.821), followed by sepal length (30.8%, -0.720), sample size (15.2%, -0.506), sepal width (11.3%, 0.436), width-position ratio (2.5%, -0.206), and contour R² (0.2%, 0.056). The dominance of tip angle quantitatively validates the classical connoisseurship distinction between ‘jian-chang’ (slender tip, low angle) and ‘tou-yuan’ (rounded head, high angle).

## 4 Discussion

### 4.1 Geometric resolution of extreme sepal morphotypes in 2D morpho space

The present study demonstrates that two-dimensional morphometric representations can capture and quantify some of the more distinct regions of floral shape variation in *C. goeringii*, although classification performance was not uniform across the traditional categories (Murphy et al., 2024). The 2D shape space does not provide uniform discriminatory power across all traditional categories, but preferentially separates morphotypes occupying well-defined geometric extremes (James et al., 2013). In this context, Zhu-ye-ban was the most distinguishable class, in terms of the highly elongated and narrowly tapered morphology and he-ban was identified with relatively lower sensitivity but high precision. This trend suggests that while lesser sensitivity shows significant overlap with other established categories, compact morphotypes may be easier to differentiate in the current feature space. These patterns indicate that the discriminative capacity of 2D morphometrics is fundamentally governed by the degree of separation in underlying shape space, rather than categorical identity alone. Although linear boundaries can distinguish relatively distinct morphotypes, significant overlap across designated categories restricts classification performance. Such overlap should not be seen as proof of developmental or evolutionary continuity within categories, but rather as resemblance in the measured planar morphology (Spani et al., 2025).

These findings directly affect digital cataloguing systems and orchid germplasm management from an applied perspective. Currently, large-scale orchid repositories rely mostly on visual recording and expert judgement, both of which are subjective by nature and challenging to standardize across institutions and time (Şeker, 2022). In contrast, the proposed 2D morphometric framework provides a cost-efficient and reproducible screening approach that could potentially support standardized digital recording and preliminary morphological screening of germplasm, while retaining expert assessment for cases that are ambiguous in the measured 2D feature space (Gargiulo et al., 2025; Spani et al., 2025). However, rather than being a proven stand-alone authentication system at this point, the framework should be viewed as a quantitative phenotyping and decision-support method.

### 4.2 Morphological overlap limits two-dimensional discrimination

The study’s primary finding is that shui-xian-ban consistently performed worst in classification, suggesting that two-dimensional descriptors alone cannot reliably distinguish its morphological traits from those of nearby sepal types. Rather than forming a distinct cluster in morphometric space, shui-xian-ban occupied an intermediate position, overlapping predominantly with mei-ban along the second discriminant axis and, to a lesser extent, with zhu-ye-ban along the first discriminant axis. This trend indicates that reduced classification accuracy is associated with substantial overlap in the measured planar shape descriptors. Nevertheless, the current data is unable to determine whether this overlap is due to variations in other unmeasured floral traits, limits of the chosen descriptors, or ongoing biological variation.

The observed overlap is compatible with the core assumptions of geometric morphometrics, where biological forms are commonly distributed along continuous shape gradients instead of discrete categories (Bookstein, 1996; Tatsuta et al., 2018). When morphological variation forms a continuum with significant overlap across classes, LDA’s performance necessarily declines since it creates linear decision boundaries that maximise separation among preset categories (James et al., 2013). Consequently, the ambiguity observed between mei-ban and shui-xian-ban is more likely to reflect the intrinsic organization of floral shape variation than methodological limitation.

Additionally, these quantitative data offer an objective interpretation of conventional horticultural classification. Classical descriptions of *C. goeringii* identify intermediate floral forms that blend traits of shui-xian-ban and mei-ban, demonstrating how these categories have traditionally been regarded as part of a progressive morphological transition rather than as entirely distinct entities (Hernández-Mejía et al., 2024). Therefore, our morphometric study does not prove that the predefined traditional categories represent developmental or evolutionary continuum, but rather gives a quantitative description of where these categories overlap in the observed feature space (Yang et al., 2026). Instead, the planar descriptors discussed here should be used to interpret the observed overlap as a limitation of category separation. This distinction is crucial because, whereas the current study assesses their correlation with quantifiable two-dimensional morphology, traditional horticultural categories are expert-defined phenotypic designations. The incorporation of additional measurable dimensions may provide complementary information for resolving complex morphological variation. Recent work on *Dendrobium officinale* polysaccharides demonstrates how integrating structural, physicochemical, and functional properties can provide a more comprehensive interpretation of biological variation (Fu et al., 2026). Similarly, advanced three-dimensional imaging approaches have shown that high-resolution, spatially resolved visualization can reveal structural information that cannot be adequately captured by conventional two-dimensional measurements (Qian et al., 2025).

### 4.3 Three-dimensional architecture as a potential limitation of planar phenotyping

The poor depiction of three-dimensional flower architecture in the current planar imaging framework may be partially responsible for the decreased discrimination of shui-xian-ban. However, it is impossible to determine how three-dimensional properties contributed to the reported classification errors from the available data because they were not directly quantified. Therefore, characteristics like qi-dou, which require curvature and spatial posture, are likely sources of information that aren’t shown in the current two-dimensional outlines (Aceto and Gaudio, 2011). The main reason for this is that this attribute cannot be adequately described as a planar curvature. Consequently, morphotypes with identical two-dimensional forms but differing three-dimensional topologies become difficult to differentiate (Zelditch et al., 2012).

This conclusion is further supported by the organ-specific misclassification patterns. While petals were more often assigned to mei-ban, the lateral sepals of shui-xian-ban were primarily identified as zhu-ye-ban. This suggests that different floral organs retain different proportions of diagnostic information following two-dimensional projection. These conclusions are disproportionately represented. This explanation is compatible with the current understanding of orchid floral development, in which sepals, petals, and the labellum follow unique developmental trajectories and contribute differently to overall floral design (Aceto and Gaudio, 2011; Mondragón-Palomino and Theißen, 2009). Different floral organs may retain varying quantities of category-associated information in the two-dimensional representation, according to the organ-specific misclassification patterns. Nevertheless, these patterns do not prove that the observed mistakes are caused by three-dimensional architecture. Although the current study was not intended to examine developmental mechanisms, this interpretation is compatible with the fact that sepals and petals differ in shape and developmental pattern.

### 4.4 Point density as a processing-dependent morphometric descriptor

This study’s key methodological discovery was that the point-density descriptor reduced direct reliance on the data while producing classification performance equivalent to the raw sample-size variable. This implies that in the current dataset, point density might offer a helpful scale-adjusted image-derived descriptor. However, picture quality, contour extraction, smoothing, and resampling techniques all have an impact on boundary-point counts in addition to specimen shape. Eliminating the raw sample size variable consistently decreased classification performance, even though it had a substantial correlation with scan length. This suggests that contour complexity contributes to discrimination beyond basic size effects. Local boundary complexity was shown to be an informative feature of sepal morphology by substituting normalized descriptor point density for sample size, which significantly reduced multicollinearity while preserving equivalent classification performance. This finding emphasizes how crucial it is to separate acquisition-dependent variables from biologically meaningful geometric descriptors when feature engineering (Mitteroecker and Gunz, 2009; Zelditch et al., 2012). In quantitative morphometric research, similar normalization techniques have been suggested to enhance model interpretability while reducing predictor redundancy. Accordingly, point density should be considered an exploratory image-derived descriptor rather than an independently validated biological measure of contour complexity. Therefore, it is not appropriate to consider the observed correlation between point density and conventional sepal categories as direct proof of biological contour complexity. Descriptor stability across standardized processing parameters and regulated imaging resolutions should be assessed in future research.

### 4.5 Implications for germplasm digitization and phenotypic screening

Molecular or provenance-based cultivar identification is complemented, not replaced, by the phenotypic information provided by the morphological features that are prioritized in traditional horticultural assessment. The current process offers a potential framework for standardized phenotypic recording of *C. goeringii* germplasm by translating certain qualitative floral characteristics into repeatable quantitative data. By converting qualitative two-dimensional morphology of the sampled floral organs into reproducible quantitative measurements, the proposed morphometric pipeline offers an objective phenotypic framework that complements existing molecular passport data and promotes standardized recording of *C. goeringii* germplasm (Aubry, 2019; Halewood et al., 2018).

The observed classification patterns suggest that a hierarchical decision-support strategy may be worth evaluating in future studies. While overlapping categories like mei-ban and shui-xian-ban might benefit from more phenotypic data or expert assessment, automated preliminary assignment might be possible for morphotypes that are well separated in the current feature space. However, rather than being a verified stand-alone authentication system, the current classification framework should be viewed as a quantitative decision-support method. We did not create a formal expert-referral mechanism, an abstention/rejection criterion, or calibrated posterior-probability thresholds. Therefore, prior to practical deployment, independent validation across cultivars, growing sites, seasons, developmental phases, and image-acquisition circumstances will be necessary. Future research should investigate uncertainty-based referral strategies and compare model effectiveness with blinded expert evaluation. Routine automated classification can be applied to morphotypes with high discrimination accuracy, specifically zhu-ye-ban. Intermediate categories such as mei-ban and shui-xian-ban should be flagged for expert verification due to their diagnostic features beyond two-dimensional morphology. This hybrid process combines the power of digital phenotyping with the reliability of expert judgement, reducing subjectivity but acknowledging the biological limitations of planar image analysis (Minervini et al., 2015; Yang et al., 2026).

However, the pipeline’s quantitative descriptors such as tip angle, length-to-width ratio, and relative width position may offer a standardised phenotypic lexicon for characterising germplasm in the future. Although the current dataset does not establish correlations between these descriptors and genetic, breeding, or functional features, their integration with further morphological, anatomical, molecular, or environmental information could be investigated in other studies (Chen et al., 2022; Crossa et al., 2021; Prakash et al., 2026).

### 4.6 Limitations and future directions

Although the proposed pipeline demonstrated robust performance for quantifying two-dimensional sepal morphology, several limitations should be acknowledged. First, the analysis was based on planar images of inherently three-dimensional floral organs; consequently, diagnostic traits associated with curvature, depth, and spatial architecture (e.g., *qi-dou*) could not be fully represented. Despite the promising solutions offered by three-dimensional imaging techniques such as structured-light scanning, photogrammetry and point-cloud reconstruction, they do not offer the same accessibility for routine and large-scale germplasm characterization due to higher acquisition costs and computational requirements (Paulus, 2019; Yang et al., 2026). An important limitation of the present study is that the analysis was based exclusively on two-dimensional outlines of detached floral organs. As a result, three-dimensional features like thickness, depth-to-width ratios, curvature, hooding, and hood volume were not measured. These characteristics might influence conventional evaluations of floral appearance, but their influence cannot be immediately deduced from the current categorization mistakes. To test this theory, more research integrating two-dimensional morphometrics with multi-view or depth-resolved three-dimensional imaging would be needed.

Second, only geometric descriptors obtained from two-dimensional morphology of the sampled floral organs were taken into account by the current system. Color, texture, venation, and surface features are also included in the traditional assessment of *C. goeringii*, and these factors may significantly aid in the identification of intermediate morphotypes. Thus, combining geometric morphometrics with multimodal phenotypic data may enhance the ability to distinguish between intermediate morphotypes; however, this needs to be investigated empirically (Gibert et al., 2025; Sheikh et al., 2024; Spani et al., 2025). The present morphometric framework captures only one component of floral phenotype, namely planar sepal geometry. Three-dimensional curvature, posture, texture, pigmentation, scent, flowering behavior, and other physiological or biochemical traits that cannot be deduced from 2D contour measurements may also be considered in traditional orchid evaluation. Therefore, it is not appropriate to interpret morphological similarity or separation seen in this study as proof of more general physiological, biochemical, or functional similarities amongst cultivars. In order to create a thorough digital framework for the objective characterization, authentication, and conservation of ornamental orchid germplasm, future research should combine three-dimensional imagery with multimodal phenotyping and genomic data.

The sampling design’s hierarchical nature is another drawback. There was reliance between observations since several floral organs from flowers of the same cultivars were analyzed. Therefore, rather than being formal population-level inference, the organ-level comparisons and classification analyses should be largely viewed as exploratory assessments of morphometric variance. To measure sources of variation and assess category × organ interactions, future research should use balanced cultivar sampling and hierarchical mixed-effects models that include cultivar, plant, flower, and organ identification.

Lastly, it is still necessary to validate the classifier’s practical usefulness. A systematic expert-referral protocol, an abstention/rejection rule, or calibrated posterior-probability limits were not established in this investigation. As a result, the suggested classification scheme shouldn’t be regarded as a verified authentication method as yet. Future research should analyse uncertainty-based referral systems and use independent cultivar-level validation across sites, seasons, developmental phases, and imaging circumstances to compare automated predictions with blinded expert judgements.

## 5 Conclusions

A repeatable two-dimensional morphometric framework for quantitatively describing the conventional sepal-shape classifications of *Cymbidium goeringii* is presented in this study. The findings show that some planar morphometric descriptors offer quantifiable discriminating across a number of conventional morphotypes, especially those with more distinctive geometric features, and are linked to the current expert-assigned categories. However, significant overlap between categories-particularly between mei-ban and shui-xian-ban-indicates that the intricacy of traditional floral classification cannot be adequately captured by the current 2D measurements alone. Since these processes were not specifically investigated in this study, the observed overlap should not be taken as proof of evolutionary or developmental continuity. Similarly, neither a final representational boundary of two-dimensional phenotyping nor a verified cultivar-authentication system is established by the current results. The results should be considered solely as an exploratory quantitative assessment of morphology linked with current traditional labels because the research was based on organ-level observations obtained from numerous organs and flowers within cultivars. Future research should assess three-dimensional floral architecture, colour, texture, venation, and other phenotypic traits using suitable hierarchical statistical techniques and independent cultivar-level validation as they may offer more discriminatory information. All things considered, the suggested workflow offers a quantitative addition to conventional morphological evaluation and lays the groundwork for the future integration of multimodal phenotyping, three-dimensional imaging, and molecular or genomic data for standardized *C. goeringii* germplasm characterization.

## Data Availability

The raw data supporting the conclusions of this article will be made available by the authors, without undue reservation.

## Supplementary material

Supplementary Figure 1 Tip angle across organs by petals-shape category. Tip angle showed a consistent decreasing trend from he-ban to zhu-ye-ban across all floral organs. The clear separation between he-ban and zhu-ye-ban, together with the overlap between mei-ban and shui-xian-ban, supports the observed classification performance and highlights organ-specific variation in floral morphology.

Supplementary Figure 2 Variance inflation factor (VIF) diagnosis for the baseline model (M1). The dashed red line indicates the conventional collinearity threshold (VIF = 5.0). Two variables- scan. length (VIF = 70.63) and sample. size (VIF = 69.97)—exhibit catastrophic multicollinearity, exceeding the threshold by more than an order of magnitude. The remaining predictors show acceptable VIF values (1.42-6.21).

Supplementary Figure 3 Taper ratio distribution across four sepal-shape categories (Model 4 feature). Taper ratio is defined as width at tip position divided by width at base position; values > 1.0 indicate a flared tip relative to a narrow base (‘shou-gen fang-jiao’ trait). White diamonds mark group means.

Supplementary Figure 4 Organ-specific classification errors and morphological divergence across floral organ types. **(A)** Error rates by organ type on the independent test set (n = 274). The central sepal (-1) exhibited the lowest error rate (35.5%). The lateral petals (-4 and -5) showed the highest error rates (51.7% and 43.9%). **(B)** Stacked bar chart showing misclassification direction of shui-xian-ban samples by organ group. Lateral sepals (fu-ban, -2 and -3) predominantly drifted toward zhu-ye-ban (68.8% of errors). Petals (peng-ban, -4 and -5) predominantly misclassified as mei-ban (69.6% of errors). **(C)** Tip angle distribution by organ type within shui-xian-ban. **(D)** Length-to-width ratio (LWR) distribution by organ type within shui-xian-ban. **(E)** Tip angle distribution across organ types by sepal-shape category.

Supplementary Figure 5 Elliptic Fourier descriptor (EFD) analysis of sepal-shape variation in *Cymbidium goeringii* (n = 1,380). **(A)** Distribution of the high-frequency ratio (efd_HFR) among the four traditional sepal shape categories, showing significant difference in contour complexity **(B)** Relationship between low- and high-frequency harmonic energies, illustrating distinct frequency-domain distributions among sepal-shape categories. **(C)** Correlation between point density and efd_HFR, indicating that harmonic complexity captures information largely independent of contour sampling density. **(D)** Fourier-based shape features differ significantly between categories when typical harmonic descriptors (H2, H3, and HFR) are compared. Overall, the point-density model outperformed EFD-derived variables in LDA classification performance, suggesting that basic contour-based descriptors were enough for differentiating sepal forms in this dataset.
