# Supplementary material for: Quantitative morphometric analysis reveals morphological overlap among traditional sepal types in Cymbidium goeringii
Source: Front Plant Sci. 2026 Oct 28;17:1947160. doi: 10.3389/fpls.2026.1947160 (PMC13752069; doi:10.3389/fpls.2026.1947160)
Supplement: Supplementary file 1 [file Table2.docx]

**Supplementary tables**

**Table S2** Variance inflation factor (VIF) diagnosis for the baseline model (M1).

| **Variable** | **VIF** | **Assessment** |
| --- | --- | --- |
| scan. length | 70.63 | Severe (>> 5.0) |
| sample. size | 69.97 | Severe (>> 5.0) |
| scan. width | 6.21 | Moderate (> 5.0) |
| tip_ angle | 5.81 | Moderate (> 5.0) |
| width_ pos_ ratio_ obs | 1.53 | Acceptable |
| R. square | 1.42 | Acceptable |

**Note:** VIF computed as 1 / (1 - R²_j) from full-model regression. Threshold for concern = 5.0.

**Table S3** Comparison of four LDA models with different feature-engineering strategies.

| **Model** | **CV_ Accuracy** | **CV_ Kappa** | **CV_ Kappa_ SD** | **Test_ Bal_ Acc** | **Shui Xian_ Sens** |
| --- | --- | --- | --- | --- | --- |
| M1_Baseline | 0.5934 | 0.4502 | 0.0760 | 0.7098 | 0.3906 |
| M2_NoSampleSize | 0.5703 | 0.4190 | 0.0871 | 0.6959 | 0.3214 |
| M3_PointDensity | 0.5855 | 0.4404 | 0.0782 | 0.7106 | 0.3538 |
| M4_TaperProxies | 0.5725 | 0.4212 | 0.0857 | 0.7112 | 0.3793 |

**Note:** CV = stratified 5-fold cross-validation; SD = standard deviation; Bal_ Acc = balanced accuracy; ShuiXian_ Sens = sensitivity for shui-xian-ban. M3 is recommended for practical germplasm screening.

**Table S4** Representative misclassified test-set samples.

| **Image** | **Cultivar** | **Actual** | **Predicted** | **Error type** | **Key traits** |
| --- | --- | --- | --- | --- | --- |
| CGH0005-3 | CGH | He-ban | Shui-xian-ban | He→ Shui-xian | Moderate tip_ angle (66.1°), low width_ pos (0.543) |
| CYS0004-1 | CYS | Zhu-ye-ban | Shui-xian-ban | Zhu-ye→ Shui-xian | Elongated (3.14 cm), moderate tip (61.1°), distal width |
| DXH0004-3 | DXH | He-ban | Shui-xian-ban | He→ Shui-xian | Moderate length (2.41 cm), low tip (61.1°), narrow width |
| DXM0007-2 | DXM | Shui-xian-ban | Mei-ban | Shui-xian→Mei | Broad (1.41 cm), high tip (67.1°), resembles mei-ban |
| Longzi0001-2 | Longzi | Shui-xian-ban | Zhu-ye-ban | Shui-xian→ Zhu-ye | Very elongated (3.71 cm), acuminate (58.4°), transitional form |
| XSM0002-5 | XSM | Shui-xian-ban | Mei-ban | Shui-xian→Mei | Compact (1.93 cm), very high tip (80.9°), all 5 images misclassified |
| Heshi0001-5 | Heshi | Mei-ban | He-ban | Mei→ He | Extremely high tip (104.4°), broad (1.28 cm), rounded contour |
| JY0002-1 | JY | Mei-ban | Shui-xian-ban | Mei→ Shui-xian | Elongated (3.29 cm), low tip (55.1°), mei-xing shui-xian form |

**Note:** Error type indicates actual → predicted category. Cultivar abbreviations: CGH = Cui Gai He, CYS = Cui Yi Su, DXH = Da Xiang He, DXM = Da Xing Mei, XSM = Xi Shen Mei, JY = Ji Yuan.

**Table S5** Descriptive statistics of morphometric traits across organ types within shui-xian-ban.

| **Organ** | **tip_angle (mean ± SD)** | **LWR (mean ± SD)** | **width (mean ± SD)** | **n** |
| --- | --- | --- | --- | --- |
| Central_ sepal | 57.7 ± 8.0 | 2.612 ± 0.415 | 1.191 ± 0.190 | 72 |
| Left_ lateral | 55.4 ± 8.7 | 2.792 ± 0.503 | 1.147 ± 0.177 | 73 |
| Left_ petal | 72.7 ± 8.7 | 1.982 ± 0.206 | 1.074 ± 0.186 | 73 |
| Right_ lateral | 54.7 ± 8.9 | 2.799 ± 0.507 | 1.147 ± 0.180 | 73 |
| Right_ petal | 73.6 ± 8.7 | 1.958 ± 0.198 | 1.079 ± 0.180 | 72 |

**Note:** Organ type identified from filename suffix: -1 = central sepal, -2/-3 = left/right lateral sepals, -4/-5 = left/right petals. All values from shui-xian-ban category only.
